# Effects of airway pressure release ventilation on lung physiology assessed by electrical impedance tomography in patients with early moderate-to-severe ARDS

**DOI:** 10.1186/s13054-023-04469-8

**Published:** 2023-05-08

**Authors:** Ruiting Li, Yongran Wu, Hongling Zhang, Azhen Wang, Xin Zhao, Shiying Yuan, Le Yang, Xiaojing Zou, You Shang, Zhanqi Zhao

**Affiliations:** 1 Department of Critical Care Medicine, Union Hospital, Tongji Medical College, Huazhong University of Science and Technology, Wuhan, Hubei China; 2 Department of Emergency Medicine, Tongji Hospital, Tongji Medical College, Huazhong University of Science and Technology, Wuhan, Hubei China; 3 Department of Biomedical Engineering, Fourth Military Medical University, Xi’an, China; 4 Institute of Technical Medicine, Furtwangen University, Villingen-Schwenningen, Germany

**Keywords:** Acute respiratory distress syndrome, Airway pressure release ventilation, Electrical impedance tomography, Heterogeneity, Ventilation/perfusion matching, Ventilator-induced lung injury

## Abstract

**Objective:**

The aim of this study was to investigate the physiological impact of airway pressure release ventilation (APRV) on patients with early moderate-to-severe acute respiratory distress syndrome (ARDS) by electrical impedance tomography (EIT).

**Methods:**

In this single-center prospective physiological study, adult patients with early moderate-to-severe ARDS mechanically ventilated with APRV were assessed by EIT shortly after APRV (T0), and 6 h (T1), 12 h (T2), and 24 h (T3) after APRV initiation. Regional ventilation and perfusion distribution, dead space (%), shunt (%), and ventilation/perfusion matching (%) based on EIT measurement at different time points were compared. Additionally, clinical variables related to respiratory and hemodynamic condition were analyzed.

**Results:**

Twelve patients were included in the study. After APRV, lung ventilation and perfusion were significantly redistributed to dorsal region. One indicator of ventilation distribution heterogeneity is the global inhomogeneity index, which decreased gradually [0.61 (0.55–0.62) to 0.50 (0.42–0.53), *p* < 0.001]. The other is the center of ventilation, which gradually shifted towards the dorsal region (43.31 ± 5.07 to 46.84 ± 4.96%, *p* = 0.048). The dorsal ventilation/perfusion matching increased significantly from T0 to T3 (25.72 ± 9.01 to 29.80 ± 7.19%, *p* = 0.007). Better dorsal ventilation (%) was significantly correlated with higher PaO_2_/FiO_2_ (r = 0.624, *p* = 0.001) and lower PaCO_2_ (r = -0.408, *p* = 0.048).

**Conclusions:**

APRV optimizes the distribution of ventilation and perfusion, reducing lung heterogeneity, which potentially reduces the risk of ventilator-induced lung injury.

**Supplementary Information:**

The online version contains supplementary material available at 10.1186/s13054-023-04469-8.

## Introduction

Airway pressure release ventilation (APRV) is a highly effective strategy improving lung recruitment and oxygenation in clinical studies, but its effects on lung injury and mortality is debatable [1, 2]. Animal studies revealed that APRV could normalize post-injury heterogeneity and reduce the risk of ventilator-induced lung injury (VILI) [3, 4]. However, the physiological effects of APRV on patients with acute respiratory distress syndrome (ARDS) have yet to be thoroughly investigated due to the unavailability of point-of-care evaluation method.

Electrical impedance tomography (EIT), a noninvasive, bedside, and radiation-free technique, has been proposed as a valid method monitoring lung ventilation and perfusion [5]. To uncover the underlying physiological mechanism, we assessed the impact of APRV on lung ventilation and perfusion in patients with early moderate-to-severe ARDS by EIT.

## Methods

### Study design

We conducted a prospective, observational study in the general ICU of Union Hospital, Tongji Medical College, Huazhong University of Science and Technology, Wuhan, China, from March 2022 to June 2022. The study was approved by the Institutional Research and Ethics Committee of Union Hospital (NO. 2022–0048). Written informed consents were obtained from the patients’ legal representatives. The study was registered before enrollment at chictr.org.cn (ChiCTR2200057638).

### Patients

The inclusion criteria were moderate-to-severe ARDS patients (defined as PaO_2_/ FiO_2_ ≤ 200 mmHg with positive end-expiratory pressure ≥ 5 cmH_2_O according to the Berlin definition [6]), endotracheal mechanical ventilation ≤ 48 h before enrollment, and expected to require continuous invasive mechanical ventilation ≥ 72 h. The exclusion criteria are provided in Additional file 1: Table S1.

### Study protocol

We screened all adult, mechanically ventilated patients. Those patients who fulfilled the criteria for ARDS were submitted to a stabilization phase during which they were ventilated with volume-controlled, assist/control mode in accordance with the recommendations of the ARDS Network [7] (detailed procedures refer to Additional file 1). At the end of this stabilization period, patients were included if they met the inclusion and exclusion criteria, and baseline respiratory mechanics were measured. Then all patients were ventilated in APRV mode (C500 Infinity ventilator, Dräger, Germany). Patients were deeply sedated and, if necessary, paralyzed to abrogate spontaneous breathing if PaO_2_/FiO_2_ < 150 mmHg, whereas spontaneous breathing was permitted in patients with PaO_2_/FiO_2_ ≥ 150 mmHg, respiratory rate less than 35 bpm, and no significant patient-ventilator asynchrony [8, 9]. Every patient maintained normovolemia during APRV.

Settings of APRV were standardized for all patients as follows. (1) Initially, high airway pressure (P_High_) was set at the plateau pressure from volume control ventilation not to exceed 30 cmH_2_O and low airway pressure (P_Low_) was set at 0 cm H_2_O. The release time (T_Low_) was set at 0.3 to 0.6 s with I:E ratio 9:1 and adjusted to achieve an end-expiratory flow rate equal to 50–75% of the peak expiratory flow rate. (2) If patients develop SpO_2_ < 90%, three options are available (detailed method refer to Additional file 1): increase P_High_ by 2 cmH_2_O until a maximum of 30 cmH_2_O, increase the time of high airway pressure (T_High_) by 0.5 s (with an unaltered T_Low_, increased T_High_ implied an increased I:E ratio and a decreased respiratory rate), and increase FiO_2_. (3) If patients develop PaCO_2_ > 60 mmHg, T_High_ can be decreased to achieve greater mandatory respiratory rate, or P_High_ can be increased by 2 cmH_2_O until a maximum of 30 cmH_2_O. (4) If the PaO_2_/FiO_2_ does not improve or remains below 150 mmHg, prone positioning will be considered. (5) If refractory hypoxemia persists, recruitment maneuver and extracorporeal membrane oxygenation will be considered.

EIT assessment (detailed procedures refer to Additional file 1, Figure S1) was administered shortly after APRV (T0), and 6 h (T1), 12 h (T2), and 24 h (T3) after APRV initiation. Parameters of ventilator, respiratory mechanics, and hemodynamics were also recorded at T0, T1, T2, and T3 before EIT assessment. Arterial blood gases analysis was recorded at T0 and T3.

### Study outcomes

The primary endpoint of the study was regional tidal volume distribution after 24 h of APRV. The second endpoints were global inhomogeneity (GI) index, center of ventilation (CoV), regional perfusion distribution, dead space-EIT (%), shunt-EIT (%), Ventilation/perfusion (V/Q) matching (%), and clinical variables related to respiratory and hemodynamic condition.

### Statistical analysis

Statistical analyses were computed with GraphPad Prism 9.0 (GraphPad Software, San Diego, CA, USA). Continuous variables were summarized as mean ± standard deviation or median (25–75th), as appropriate. A paired t-test was used to compare the paired data. Repeated measures ANOVA or Friedman test was applied with post-hoc Bonferroni’s or Dunn’s multiple comparisons to compare the data obtained at each time points, as appropriate. Correlation between continuous variables was assessed by the nonparametric Spearman correlation. A level of *p* value less than 0.05 (two-tailed) was considered as statistically significant.

## Results

Twelve ARDS patients (9 male, 3 female) were included in the study. Mean age was 53.67 ± 12.37 years. There were 5 (42%) patients with severe ARDS and 7 (58%) patients with moderate ARDS. More detailed clinical characteristics of the study population were presented in Additional file 1: Table S2. During APRV, no patient developed barotrauma (pneumothorax, mediastinal emphysema, or subcutaneous emphysema), and all patients were in supine position.

**Fig. 1:**
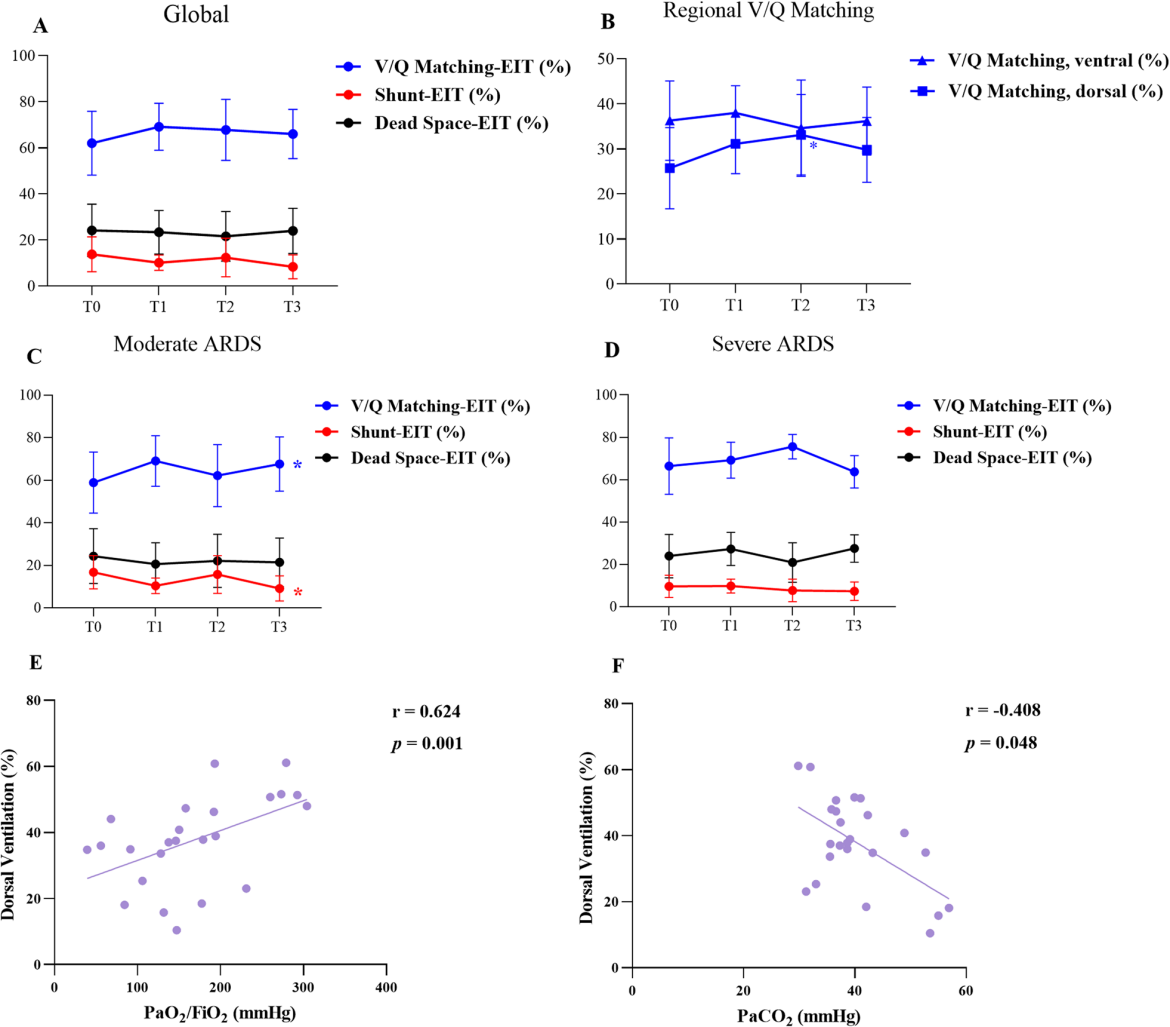
Evolution of global shunt-EIT (%), dead space-EIT (%), and V/Q matching (%) at T0, T1, T2 and T3 (**A**). Evolution of V/Q matching (%) in ventral and dorsal region at T0, T1, T2 and T3 (**B**). Subgroup analysis of V/Q matching (%), shunt-EIT (%), and dead space-EIT (%) evolution at T0, T1, T2, and T3 (**C**, **D**). Patients were divided into two subgroups based on the severity of ARDS: moderate ARDS with seven patients (**C**) and severe ARDS with five patients (**D**). Better dorsal ventilation (%) was significantly correlated with higher PaO_2_/FiO_2_ (**E**) and lower PaCO_2_ (**F**). V/Q, ventilation/perfusion; EIT, Electrical impedance tomography; ARDS, acute respiratory syndrome. T0: shortly after APRV; T1, T2, and T3: 6 h, 12 h, and 24 h after APRV application. *p < 0.05

Data from EIT-based measurements showed that the regional lung ventilation and perfusion significantly redistributed from the ventral to dorsal region following ARPV application (Table 1). The median GI index of ventilation progressively decreased [0.61 (0.55–0.62) to 0.50 (0.42–0.53), *p* < 0.001], and CoV gradually changed towards dorsal regions (43.31 ± 5.07 to 46.84 ± 4.96%, *p* < 0.05). Patients trend to have an increased global V/Q matching (%) (63.41 ± 13.47 to 68.58 ± 11.70%) and decreased shunt-EIT (%) (13.80 ± 7.53 to 8.39 ± 5.18%) from T0 to T3, but not significantly; however, the dorsal V/Q matching (%) increased significantly (25.72 ± 9.01 to 29.80 ± 7.19%, *p* = 0.007) (Fig. 1). In subgroup analysis, patients with moderate ARDS had a significant increase in global V/Q matching (%) (58.88 ± 14.34 to 67.60 ± 12.75%, *p* = 0.019) and a decrease in global functional shunt (16.78 ± 7.81 to 9.16 ± 5.92%, *p* = 0.040), whereas patients with severe ARDS had no improvement (Fig. 1).

**Table 1:** Electrical impedance tomography data analysis of selected physiologic variables, respiratory parameters, and hemodynamic parameters at the four different time points

Variables	T0	T1	T2	T3	*P* value
*EIT data*					
Ventilation distribution, ventral (%)	68.87 ± 10.94	61.04 ± 12.80^a^	58.66 ± 12.51^a^	55.83 ± 13.48^a^	< 0.001
Ventilation distribution, dorsal (%)	31.13 ± 10.94	38.96 ± 12.80^a^	41.34 ± 12.51^a^	44.17 ± 13.48^a^	< 0.001
Ventilation distribution, ROI 1 (%)	16.01 (9.93–22.39)	10.99 (7.40–16.66)^a^	12.95 (9.13–15.35)	12.05 (7.30–15.68)^a^	0.009
Ventilation distribution, ROI 2 (%)	52.12 ± 5.54	48.81 ± 9.34	46.07 ± 8.73	42.88 ± 9.24^a^	0.002
Ventilation distribution, ROI 3 (%)	26.62 ± 10.21	33.42 ± 9.37	36.56 ± 10.75^a^	35.51 ± 11.11^a^	0.001
Ventilation distribution, ROI 4 (%)	4.52 ± 3.58	5.56 ± 6.40	4.78 ± 5.57	8.66 ± 5.32^a^	0.027
Perfusion distribution, ventral (%)	57.75 (53.81–67.97)	56.20 (52.72–61.52)	57.37 (47.63–63.24)	49.62 (46.80–58.74)^a^	0.013
Perfusion distribution, dorsal (%)	42.25 (32.03–46.19)	43.80 (38.48–47.28)	42.63 (36.76–52.37)	50.38 (41.26–53.20)^a^	0.013
Perfusion distribution, ROI 1 (%)	15.86 ± 8.62	12.61 ± 4.54	12.54 ± 5.10	13.11 ± 4.91	0.405
Perfusion distribution, ROI 2 (%)	46.76 ± 9.18	44.43 ± 4.83	42.87 ± 5.64	38.99 ± 6.00^a^	0.009
Perfusion distribution, ROI 3 (%)	36.32 (27.99–39.37)	37.82 (29.37–42.87)	36 (30.81–42.19)	39.47 (33.76–43.95)^a^	0.037
Perfusion distribution, ROI 4 (%)	5.93 ± 3.16	6.45 ± 3.04	7.40 ± 3.71	9.00 ± 3.29^a b^	0.005
GI index-ventilation	0.61 (0.55–0.62)	0.54 (0.51–0.61)	0.53 (0.47–0.55)^a^	0.50 (0.42–0.53)^a^	< 0.001
Center of Ventilation (%)	43.31 ± 5.07	45.09 ± 4.88	46.05 ± 4.63	46.84 ± 4.96^a^	0.048
Shunt (%)	13.80 ± 7.53	10.15 ± 3.34	12.39 ± 8.37	8.39 ± 5.18	0.059
Shunt, ventral (%)	4.29 (1.94–7.03)	2.87 (1.07–5.66)	3.39 (1.79–6.62)	2.30 (1.02–3.57)	0.348
Shunt, dorsal (%)	7.38 (4.99–13.01)	6.41 (4.94–8.75)	5.60 (3.00–10.49)	4.80 (2.80–8.38)	0.458
Dead space (%)	23.92 (14.71–31.43)	25.54 (15.37–29.57)	18.19 (15.02–28.31)	26.81 (19.34–31.08)	0.777
Dead space, ventral (%)	8.30 ± 2.40	8.49 ± 2.45	9.25 ± 2.67	7.13 ± 2.06	0.906
Dead space, dorsal (%)	6.99 (4.70–9.13)	7.95 (4.60–10.64)	7.26 (4.40–9.86)	8.67 (6.27–13.02)	0.552
V/Q matching (%)	63.41 ± 13.47	69.13 ± 10.18	67.76 ± 13.25	68.58 ± 11.70	0.258
V/Q matching, ventral (%)	35.89 (30.51–43.33)	38.28 (34.23–42.30)	34.07 (27.65–43.14)	34.43 (31.38–36.78)	0.682
V/Q matching, dorsal (%)	25.72 ± 9.01	31.13 ± 6.62	33.14 ± 8.95^a^	29.80 ± 7.19	0.007
*Respiratory parameters*					
PaO_2_	75.16 ± 17.19	–	–	116.1 ± 41.24	0.004
PaCO_2_	44.27 ± 8.58	–	–	36.78 ± 4.20	0.032
PaO_2_/FiO_2_	109.5 ± 36.44	–	–	222.2 ± 58.46	< 0.001
Mean airway pressure (cmH_2_O)	20.83 ± 2.21	20.75 ± 2.30	20.83 ± 2.13	20.50 ± 1.78	0.448
Total minute ventilation (L/min)	5.88 ± 0.90	6.14 ± 0.99	6.55 ± 1.19^a^	7.53 ± 1.22^abc^	< 0.001
Tidal volume (mL/kg PBW)	6.90 ± 0.68	7.19 ± 0.74	7.66 ± 0.74^a^	8.82 ± 0.84^abc^	< 0.001
Tidal volume (mL)	442.5 ± 32.86	461.3 ± 36.15	491.8 ± 38.8^a^	567.5 ± 60.93^abc^	< 0.001
*Driving pressure (cmH_2_O)	11.93 ± 1.73	11.40 ± 1.61	10.86 ± 2.15	10.30 ± 1.97	0.090
*Crs (mL/cmH_2_O)	32.15 (27.11–42.3)	32.40 (29.63–39.98)	38.45 (30.15–45.53)	37.95 (36.00–44.25)	0.030
*Hemodynamic parameters*					
Systolic blood pressure (mmHg)	118.3 ± 17.83	125.1 ± 16.18	125.4 ± 28.22	130.8 ± 26.98	0.624
Diastolic blood pressure (mmHg)	62 ± 9.42	64.75 ± 8.76	61.33 ± 13.9	65.67 ± 11.62	0.734
Heart rate (bpm)	103 ± 19.24	93.92 ± 17.71	90.67 ± 90.67	89.17 ± 12.48	0.185
Norepinephrine dose (μg/kg/min)	0 (0–0.246)	0 (0–0.17)	0.05 (0–0.15)	0 (0–0.15)	0.968

Data are mean ± SD or median (25–75th)

^*^The methods for calculating driving pressure and Crs are provided in Additional file 1

ROI, Region of Interest; GI, Global Inhomogeneity; V/Q, ventilation/perfusion; PaO_2_/FiO_2_, ratio of partial pressure arterial oxygen and fraction of inspired oxygen; Crs, respiratory system compliance

T0: shortly after APRV; T1, T2, and T3: 6 h, 12 h, and 24 h after APRV application

^a^vs. T0, *P* < 0.05

^b^vs. T1, *P* < 0.05

^c^vs. T2, p < 0.05

From T0 to T3, median respiratory system static compliance after APRV application increased from 32.15 (27.11–42.30) to 37.95 (36.00–44.25) mL/cmH_2_O (*p* = 0.030). Additionally, PaO_2_/FiO_2_ increased from 109.5 ± 36.44 to 222.2 ± 58.46 mmHg significantly (*p* < 0.001), and PaCO_2_ decreased from 44.27 ± 8.58 to 36.78 ± 4.20 mmHg significantly (*p* = 0.032). Better dorsal ventilation (%) was significantly correlated with higher PaO_2_/FiO_2_ (r = 0.624, *p* = 0.001) and lower PaCO_2_ (r = -0.408, *p* = 0.048) (Fig. 1). No significant difference was identified in hemodynamic variables.

## Discussion

To the best of our knowledge, this is the first study using EIT to assess the physiological effects of APRV on lung ventilation and perfusion distribution, and V/Q matching in patients with early moderate-to-severe ARDS.

This study showed that dorsal region recruited after 24 h of APRV; moreover, the lung ventilation became more homogenous according to the results of GI index and CoV, suggesting that APRV has the potential to reduce the risk of VILI. Interestingly, we observed that ventilation redistributed prior to perfusion redistribution, implying that optimized ventilation facilitated in perfusion improvement. Dorsal alveolar recruitment reduced pulmonary vascular resistance, facilitating significant improvement of dorsal perfusion and V/Q matching. On the contrary, if lung inflation caused overexpansion rather than alveolar recruitment, an increase in pulmonary vascular resistance in hyperinflated regions would also direct perfusion toward nonaerated dorsal regions, ultimately leading to increased functional shunt and dead space [10].

The number of unmatched regions is associated with ARDS severity and the risk of VILI, as well as being an independent predictor of mortality [11]. Our study showed that V/Q matching improved and functional shunt decreased significantly in the moderate ARDS subgroup after 24 h APRV. Unfortunately, no significant improvement in V/Q matching was observed in severe ARDS, which could be attributed to the lack of significant improvement in perfusion redistribution (Additional file 1: Table S3). Pulmonary microvascular thrombosis, endothelial swelling and damage, and abnormal vasocontraction could lead to significant local hypoperfusion or even vascular occlusion, resulting in increased dead space [12]. In this situation, in addition to alveolar recruitment, inhaled pulmonary vasodilators, anti-inflammatory, or anticoagulation might be used as adjunctive therapy, but the evidence is limited, necessitating additional research [13].

There are several limitations in our study. First, the sample size was limited. Second, we didn’t focus on the effects of spontaneous breathing on APRV. The effects of APRV with or without spontaneous breathing on ARDS patients are still being debated [14]. Third, EIT only provides a cross-sectional lung-region analysis, which may differ from whole-lung evaluation. Forth, cardiac output was not measured in this cohort. Fifth, we didn’t measure end expiratory lung volume to assess any variation induced by APRV. Finally, there was no control group. Future research should focus on these issues.

## Conclusions

APRV is a strategy based on pathophysiology that provides lung recruitment, stabilization, and homogeneity, potentially protecting injured lungs in patients with early moderate-to-severe ARDS.

## Supplementary Information

**Additional file 1. Methods. Table S1.** Exclusion criteria. **Table S2.** Patients’ main characteristics. **Table S3.** Subgroup analysis of ventilation and perfusion distribution evolution at T0, T1, T2, and T3. **Fig. S1.** Ventilation and perfusion measured by EIT in a representative patient at different time points.

## Data Availability

The datasets used and/or analyzed during the current study are available from the corresponding author on reasonable request.

## Abbreviations

APRV: Airway pressure release ventilation;
VILI: Ventilator-induced lung injury
ARDS: Acute respiratory distress syndrome
EIT: Electrical impedance tomography
VCV: Volume-controlled ventilation
V/Q: Ventilation/perfusion
GI: Global inhomogeneity
CoV: Center of ventilation

## References

[1] Zhou Y, Jin X, Lv Y, Wang P, Yang Y, Liang G, Wang B, Kang Y (2017). Early application of airway pressure release ventilation may reduce the duration of mechanical ventilation in acute respiratory distress syndrome. Intensive Care Med.

[2] Ibarra-Estrada MA, Garcia-Salas Y, Mireles-Cabodevila E, Lopez-Pulgarin JA, Chavez-Pena Q, Garcia-Salcido R, Mijangos-Mendez JC, Aguirre-Avalos G (2022). Use of airway pressure release ventilation in patients with acute respiratory failure due to COVID-19: results of a single-center randomized controlled trial. Crit Care Med.

[3] Kollisch-Singule M, Emr B, Smith B, Roy S, Jain S, Satalin J, Snyder K, Andrews P, Habashi N, Bates J (2014). Mechanical breath profile of airway pressure release ventilation: the effect on alveolar recruitment and microstrain in acute lung injury. JAMA Surg.

[4] Kollisch-Singule M, Emr B, Smith B, Ruiz C, Roy S, Meng Q, Jain S, Satalin J, Snyder K, Ghosh A (2014). Airway pressure release ventilation reduces conducting airway micro-strain in lung injury. J Am Coll Surg.

[5] Jimenez JV, Weirauch AJ, Culter CA, Choi PJ, Hyzy RC (2022). Electrical impedance tomography in acute respiratory distress syndrome management. Crit Care Med.

[6] Force ADT, Ranieri VM, Rubenfeld GD, Thompson BT, Ferguson ND, Caldwell E, Fan E, Camporota L, Slutsky AS (2012). Acute respiratory distress syndrome: the Berlin definition. JAMA.

[7] Acute Respiratory Distress Syndrome N, Brower RG, Matthay MA, Morris A, Schoenfeld D, Thompson BT, Wheeler A: Ventilation with lower tidal volumes as compared with traditional tidal volumes for acute lung injury and the acute respiratory distress syndrome. *N Engl J Med* 2000, 342(18):1301–1308. 10.1056/NEJM200005043421801 10793162

[8] Papazian L, Aubron C, Brochard L, Chiche JD, Combes A, Dreyfuss D, Forel JM, Guerin C, Jaber S, Mekontso-Dessap A (2019). Formal guidelines: management of acute respiratory distress syndrome. Ann Intensive Care.

[9] Tasaka S, Ohshimo S, Takeuchi M, Yasuda H, Ichikado K, Tsushima K, Egi M, Hashimoto S, Shime N, Saito O (2022). ARDS Clinical Practice Guideline 2021. J Intensive Care.

[10] Musch G, Harris RS, Vidal Melo MF, O'Neill KR, Layfield JD, Winkler T, Venegas JG (2004). Mechanism by which a sustained inflation can worsen oxygenation in acute lung injury. Anesthesiology.

[11] Spinelli E, Kircher M, Stender B, Ottaviani I, Basile MC, Marongiu I, Colussi G, Grasselli G, Pesenti A, Mauri T (2021). Unmatched ventilation and perfusion measured by electrical impedance tomography predicts the outcome of ARDS. Crit Care.

[12] Beda A, Winkler T, Wellman TJ, De Prost N, Tucci M, Vidal Melo MF (2021). Physiological mechanism and spatial distribution of increased alveolar dead-space in early ARDS: an experimental study. Acta Anaesthesiol Scand.

[13] Rialp G, Betbese AJ, Perez-Marquez M, Mancebo J (2001). Short-term effects of inhaled nitric oxide and prone position in pulmonary and extrapulmonary acute respiratory distress syndrome. Am J Respir Crit Care Med.

[14] Andrews P, Shiber J, Madden M, Nieman GF, Camporota L, Habashi NM (2022). Myths and misconceptions of airway pressure release ventilation: getting past the noise and on to the signal. Front Physiol.

