# Supplementary material for: Effects of airway pressure release ventilation on lung physiology assessed by electrical impedance tomography in patients with early moderate-to-severe ARDS
Source: Crit Care. 2023 May 8;27:178. doi: 10.1186/s13054-023-04469-8 (PMC10169478; doi:10.1186/s13054-023-04469-8)
Supplement: Supplementary file 1 — Additional file 1. Methods. Table S1. Exclusion criteria. Table S2. Patients’ main characteristics. Table S3. Subgroup analysis of ventilation and perfusion distribution evolution at T0, T1, T2, and T3. Fig. S1. Ventilation and perfusion measured by EIT in a representative patient at different time points. [file 13054_2023_4469_MOESM1_ESM.docx]

**Additional file 1**

**Effects of airway pressure release ventilation on lung physiology assessed by electrical impedance tomography in patients with early moderate-to-severe ARDS**

Ruiting Li, Yongran Wu, Hongling Zhang, Azhen Wang, Xin Zhao, Shiying Yuan, Le Yang, Xiaojing Zou, You Shang, Zhanqi Zhao

**Methods**

**Mechanical settings of volume control ventilation**

All patients must complete a “stabilization” period of volume-controlled, assist/control mode to take baseline measurements, with tidal volume 6-8 ml/kg predicted body weight. Titration of positive end expiratory pressure (PEEP) and FiO_2_ will be performed as suggested by acute respiratory distress syndrome (ARDS) network with preference for the lower PEEP/higher FiO_2_ table. The goals of mechanical ventilation were to maintain plateau pressure ≤30 cm H_2_O, pulse oximeter ≥88% or PaO_2_ ≥55 mmHg, and PaCO_2_ 35-60mmHg.

**Detailed method for optimizing oxygenation during APRV**

If patients develop SpO_2_ <90%, three options are available: increase T_High_, P_High,_ and FiO_2_. When making a decision, PaCO_2_ should be taken into account.

1.Without CO_2_ retention, increase T_High_ by 0.5 second and P_High_ by 2 cmH_2_O until a maximum of 30 cmH_2_O simultaneously.

2.With severe CO_2_ retention, increase P_High_ by 2 cmH_2_O until a maximum of 30 cmH_2_O.

3.If there was no improvement in oxygenation within 20-30 minutes despite the above adjustments, increase FiO_2_.

**Methods for calculating driving pressure and respiratory system compliance**

The formula of driving pressure (DP) was based on estimating the intrinsic PEEP present at the end of the APRV release, which was calculated as [1]:

DP = Pplat - PEEPtot

Pplat, measured from maximum pressure in the lung pressure waveform

PEEPtot, measured from lung pressure at end expiration in lung pressure waveform

Respiratory system compliance (Crs) was calculated as:

Crs = Release volume/DP

**EIT assessment methods**

EIT (electrical impedance tomography) functional images and data were generated by PulmoVista 500 (Dräger Medical, Lübeck, Germany). The EIT belt with 16 surface electrodes was placed around the thorax at the fourth intercostal space level. EIT images were continuously recorded at 20 Hz. After a baseline recording of EIT data for 5 min, we performed an end-expiratory breath hold lasting 10 s. Two seconds after the start of the occlusion, a bolus of 10 ml of 10% NaCl solution was manually injected via the central venous catheter. The bolus of saline solution, injected in less than 2 s, passes through the pulmonary circulation producing an impedance dilution curve that follows typical first-pass kinetics [1].

EIT ventilation maps were analyzed offline to average the values of five consecutive respiratory cycles. For the quantitative analysis of the ventilation and perfusion distributions by EIT, the lungs were sub-segmented into ventral and dorsal regions. Ventral regions included two regions-of-interest (ROIs): ROI 1 (ventral) and ROI 2 (mid-ventral). Dorsal region included two ROIs: ROI 3 (mid-dorsal) and ROI 4 (dorsal) [2].

Regional functional ventilation map was calculated by subtracting the end-expiration impedance from the end-inspiration impedance, which represents the local volume variation during tidal breathing. From the analysis of ventilation maps, we assessed the following measures:

1. The percentage of ventilation distribution in the respective region.

2. The global inhomogeneity (GI) [3] index was used to assess the ventilation distribution.

3. Center of ventilation (CoV) [4] was calculated to describe the distribution in the vertical (ventral-to-dorsal) axis.

Regional functional perfusion maps were derived from analyzing the slope of time-impedance curve after saline injection after removing the cardiac region from the images. Ventilated and perfused regions were defined as pixels higher than 20% maximum of the functional ventilation and perfusion maps, respectively. From the analysis of perfusion maps, four measures were calculated:

1. The percentage of perfusion distribution in the respective ROIs.

2. Dead space-EIT (%), corresponding to regions that were only ventilated.

3. Shunt-EIT (%), corresponding to regions that were only perfused.

4. V/Q matching (%), corresponding to regions that were both ventilated and perfused.

Table S1. Exclusion criteria

| - Age of <18 years |
| --- |
| - Body mass index >35 kg/m^2^ |
| - Refractory shock |
| - Pregnancy |
| - Severe cardiac dysfunction |
| - Contraindications to the use of airway pressure release ventilation (pneumothorax, severe chronic obstructive pulmonary disease, severe asthma, and intracranial hypertension) |
| - Contraindications to the use of electrical impedance tomography (e.g., chest surgical wounds dressing or presence of pacemaker) |

Table S2 Patients' main characteristics

| Variable | All Patients, n = 12 |
| --- | --- |
| Patients' characteristics |  |
| Male, n (%) | 9 (75.0) |
| Age (years) | 53.67 ± 12.37 |
| Body mass index (kg/m^2^) | 23.26 ± 3.84 |
| Predicted body weight (kg) | 64.50 ± 5.27 |
| Comorbidities, n (%) |  |
| Hypertension | 4 (33.3) |
| Diabetes | 4 (33.3) |
| ARDS etiology, n (%) |  |
| Pneumonia | 6 (50.0) |
| Sepsis | 4 (33.3) |
| Major surgery | 2 (16.7) |
| ARDS severity, n (%) |  |
| Moderate | 7 (58.3) |
| Severe | 5 (41.7) |
| ARDS morphology, n (%) |  |
| Focal | 6 (50%) |
| Non-focal | 6 (50%) |
| APACHE II score at ICU admission | 21.58 ± 7.56 |
| SOFA score at ICU admission | 8.50 ± 3.61 |
| MV duration before enrollment (hours) | 25.13 ± 10.76 |
| Gas exchange and mechanics at enrollment |  |
| PaO_2_/FiO_2_ (mmHg) | 98.58 ± 30.89 |
| PaCO_2_ (mmHg) | 44.58 ± 8.35 |
| Static respiratory system compliance (mL/cmH_2_O) | 32.92 ± 6.47 |
| Ventilator settings of volume control ventilation |  |
| FiO_2_ (%) | 67.08 ± 19.36 |
| Positive end-expiratory pressure (cm H_2_O) | 10 (10–13.5) |
| Tidal volume (mL/kg predicted body weight) | 6.73 ± 0.67 |
| Respiratory rate (breaths/min) | 21.25 ± 3.49 |

APACHE-II, acute physiology and chronic health evaluation II; ICU, intensive care unit; SOFA, sequential organ failure assessment; PaO_2_/FiO_2_, ratio of partial pressure arterial oxygen and fraction of inspired oxygen; MV, mechanical ventilation

Table S3 Subgroup analysis of ventilation and perfusion distribution evolution at T0, T1, T2, and T3

| **Variables** | **T0** | **T1** | **T2** | **T3** | ***P*** value |
| --- | --- | --- | --- | --- | --- |
| Moderate ARDS |  |  |  |  |  |
| Ventilation distribution, ventral (%) | 70.60 ± 12.29 | 61.84 ± 14.26a | 61.56 ± 13.85 | 57.12 ± 14.13 | 0.055 |
| Ventilation distribution, dorsal (%) | 29.40 ± 12.29 | 38.16 ± 14.26a | 38.44 ± 13.85 | 42.88 ± 14.13 | 0.055 |
| Perfusion distribution, ventral (%) | 62.73 ± 13.24 | 58.25 ± 8.21 | 57.97 ± 7.27a | 53.80 ± 5.70a | **0.027** |
| Perfusion distribution, dorsal (%) | 37.27 ± 13.24 | 41.75 ± 8.21 | 42.03 ± 7.27a | 46.20 ± 5.70a | **0.027** |
| Severe ARDS |  |  |  |  |  |
| Ventilation distribution, ventral (%) | 66.44 ± 9.47 | 59.93 ± 11.97 | 54.6 ± 10.38a | 54.01 ± 13.91a | **0.014** |
| Ventilation distribution, dorsal (%) | 33.56 ± 9.47 | 40.07 ± 11.97 | 45.4 ± 10.38a | 45.99 ± 13.91a | **0.014** |
| Perfusion distribution, ventral (%) | 55.29 (53.92–76.15) | 55.03 (49.78–61.11) | 55.49 (40.99–60.84) | 46.84 (46.18–54.74) | 0.408 |
| Perfusion distribution, dorsal (%) | 44.71 (23.85–46.08) | 44.97 (38.89–50.22) | 44.51 (39.16–59.01) | 53.16 (45.26–53.82) | 0.408 |

Data are mean ± SD or median (25–75th)

ARDS, Acute respiratory distress syndrome

T0: shortly after APRV; T1, T2, and T3: 6 h, 12 h, and 24 h after APRV application

^a^ vs. T0, *P*< 0.05


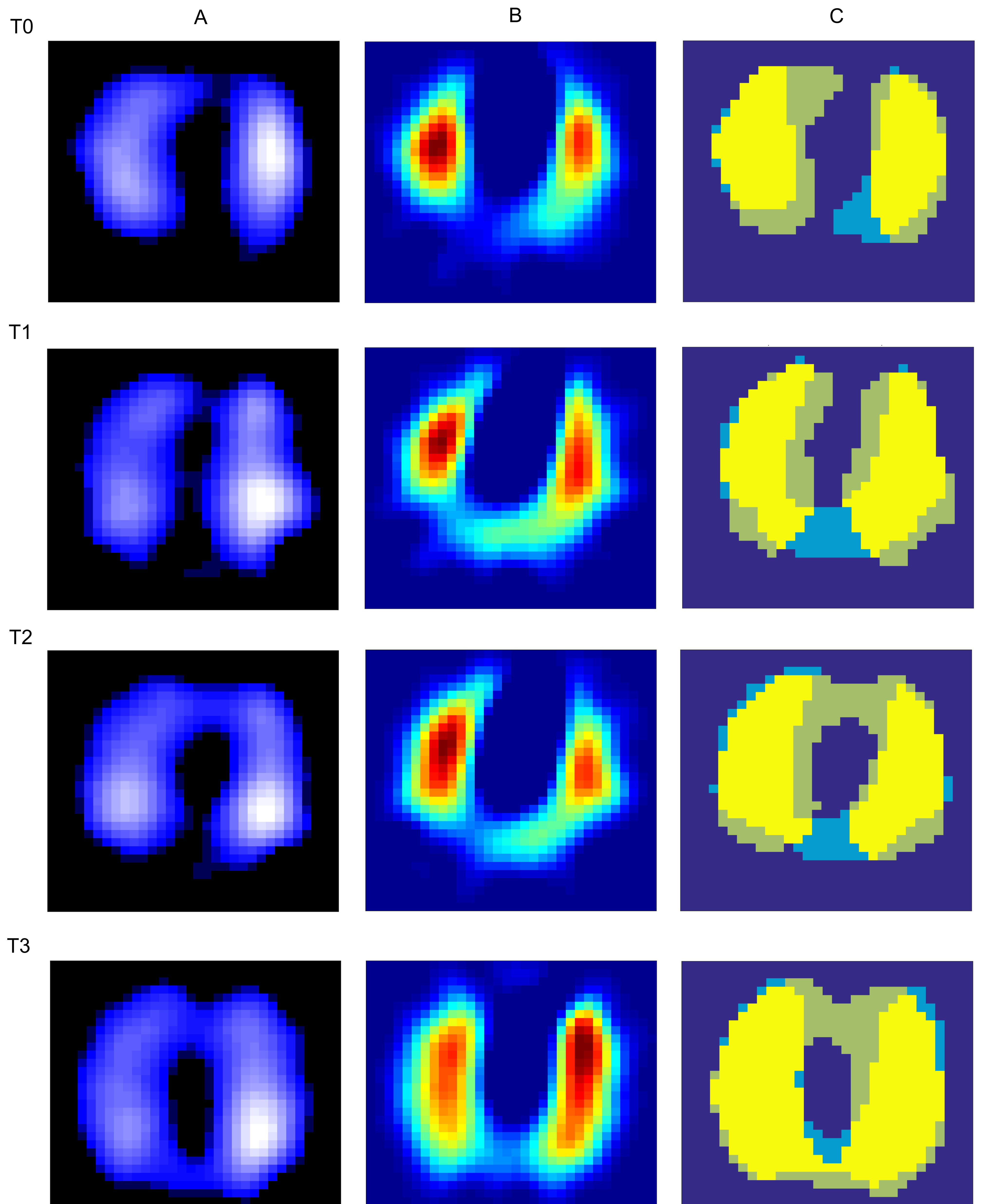


Figure S1. Ventilation and perfusion measured by EIT in a representative patient at different time points. Left map (A): image of the ventilation distribution. Middle map (B): image of perfusion distribution. Right map (C): obtained by integrating ventilation and perfusion maps: the yellow area indicates matched units which are both ventilated and perfused, while blue area indicated only perfused units and light green area indicated only ventilated units. T0: shortly after APRV; T1, T2, and T3: 6 h, 12 h, and 24 h after APRV application

**Reference**

1. He H, Long Y, Frerichs I, Zhao Z: **Detection of Acute Pulmonary Embolism by Electrical Impedance Tomography and Saline Bolus Injection**. *Am J Respir Crit Care Med* 2020, **202**(6):881-882.

2. Victorino JA, Borges JB, Okamoto VN, Matos GF, Tucci MR, Caramez MP, Tanaka H, Sipmann FS, Santos DC, Barbas CS *et al*: **Imbalances in regional lung ventilation: a validation study on electrical impedance tomography**. *Am J Respir Crit Care Med* 2004, **169**(7):791-800.

3. Zhao Z, Moller K, Steinmann D, Frerichs I, Guttmann J: **Evaluation of an electrical impedance tomography-based Global Inhomogeneity Index for pulmonary ventilation distribution**. *Intensive Care Med* 2009, **35**(11):1900-1906.

4. Frerichs I, Hahn G, Golisch W, Kurpitz M, Burchardi H, Hellige G: **Monitoring perioperative changes in distribution of pulmonary ventilation by functional electrical impedance tomography**. *Acta Anaesthesiol Scand* 1998, **42**(6):721-726
